# Retrieval of Brain Tumors by Adaptive Spatial Pooling and Fisher Vector Representation

**DOI:** 10.1371/journal.pone.0157112

**Published:** 2016-06-06

**Authors:** Jun Cheng, Wei Yang, Meiyan Huang, Wei Huang, Jun Jiang, Yujia Zhou, Ru Yang, Jie Zhao, Yanqiu Feng, Qianjin Feng, Wufan Chen

**Affiliations:** 1 School of Biomedical Engineering, Southern Medical University, Guangzhou, China; 2 School of Medical Information Engineering, Guangdong Pharmaceutical University, Guangzhou, China; University of North Carolina, UNITED STATES

## Abstract

Content-based image retrieval (CBIR) techniques have currently gained increasing popularity in the medical field because they can use numerous and valuable archived images to support clinical decisions. In this paper, we concentrate on developing a CBIR system for retrieving brain tumors in T1-weighted contrast-enhanced MRI images. Specifically, when the user roughly outlines the tumor region of a query image, brain tumor images in the database of the same pathological type are expected to be returned. We propose a novel feature extraction framework to improve the retrieval performance. The proposed framework consists of three steps. First, we augment the tumor region and use the augmented tumor region as the region of interest to incorporate informative contextual information. Second, the augmented tumor region is split into subregions by an adaptive spatial division method based on intensity orders; within each subregion, we extract raw image patches as local features. Third, we apply the Fisher kernel framework to aggregate the local features of each subregion into a respective single vector representation and concatenate these per-subregion vector representations to obtain an image-level signature. After feature extraction, a closed-form metric learning algorithm is applied to measure the similarity between the query image and database images. Extensive experiments are conducted on a large dataset of 3604 images with three types of brain tumors, namely, meningiomas, gliomas, and pituitary tumors. The mean average precision can reach 94.68%. Experimental results demonstrate the power of the proposed algorithm against some related state-of-the-art methods on the same dataset.

## Introduction

In modern hospitals, a large number of medical images are produced, diagnosed, and archived in picture archiving and communication systems every day. The use of stored visual data for clinical decision support, radiologist training, and research in medical schools would be of great clinical benefit. These demands have made CBIR an active research area in medicine. Compared with text-based image retrieval, the CBIR can search query images from a database according to their visual content. Recent years have witnessed a growing interest in CBIR for various medical images, such as MRI images [1–3], CT images [4], mammograms [5], and pathology images [6]. In this paper, we focus on developing a CBIR system for retrieving MRI images of brain tumors to assist radiologists in the diagnosis of brain tumors.

**Fig 1:**
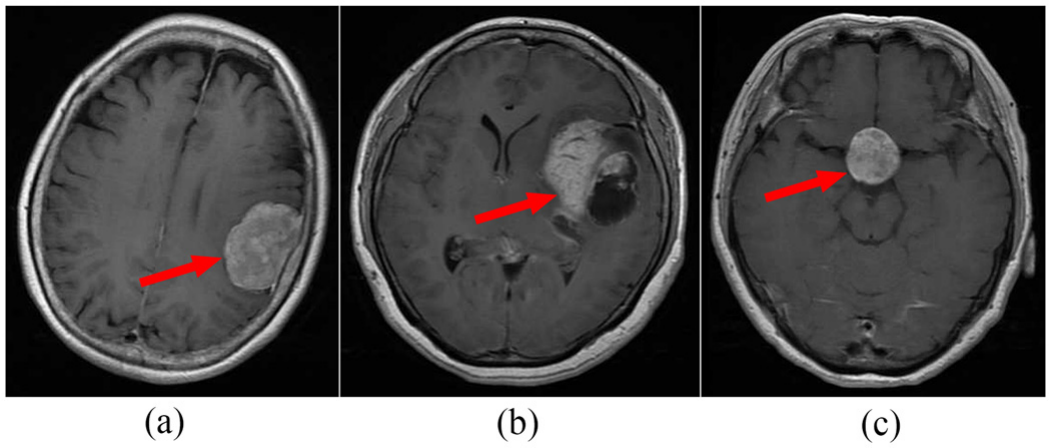
Both (a) and (b) are meningiomas, but they have very different appearances. Although (c) is a pituitary tumor, it exhibits similar appearances to (a). Red arrows indicate tumors.

Accurate diagnosis is important for the successful treatment of brain tumors. However, the diagnosis of the brain tumor is challenging because even brain tumors of the same class can have large variations in their shape, margin, size, and texture because of the severity, age, or some other factors. And conversely, tumors belonging to different pathological types may exhibit similar appearances. Example images are shown in Fig 1.

Conventional diagnoses of brain tumor images are made by human interpretation, which heavily relies on the experience of radiologists who review and analyze the characteristics of the images. Consequently, interobserver and intraobserver variability are inevitable. Nevertheless, the CBIR-based computer-aided diagnosis system can easily solve this problem in a systematic manner. When the radiologist is uncertain of the diagnosis of a brain tumor case, he can search the database of past resolved cases for images that have the most similar visual features to those of the query image. Based on the associated diagnostic information of the retrieved set, the radiologist can make a diagnostic decision.

The key factors for the development of CBIR systems of high retrieval accuracy are discriminative features and a suitable similarity/distance metric. The feature extraction is a fundamental step. First-order statistics (e.g., the mean, standard deviation, and skewness) and second-order statistics derived from gray level co-occurrence matrix, shape, and Gabor filters are frequently used low-level features [1,7–11]. Unfortunately, the power of these low-level features is limited because of the complex texture presented in the tumor region. In addition to these low-level features, there are some more effective alternatives such as bag-of-words (BoW) model [12–14] and Fisher vector (FV) [15,16], both of which aggregate local features into a single vector representation and are commonly used in the computer vision community. Briefly, BoW representations can be extracted in two steps. First, a visual vocabulary is built by clustering the feature space populated with local features extracted from image (or ROI) set. The clustering centroids are the visual words in the visual vocabulary. Second, the local features of an image are extracted and quantized, and then the image is represented as a vector of visual word occurrences. Compared with BoW, FV usually show much better performance for general image classification and retrieval tasks [15,17,18]. Moreover, FV is cheaper to compute because less visual words are required. FV represents a sample by its deviation from the generative model. The deviation is measured by calculating the gradient of the sample log-likelihood with respect to the model parameters. In this paper, in terms of brain tumor retrieval, we compared Bow and FV representations, and also verified that FV is vastly superior to BoW.

Note that medical images are distinctly different from general images, such that some kinds of local features that work well for general images may not apply to medical images. For instance, the scale-invariant feature transform (SIFT) descriptor, a well-known local feature that is typically extracted from key points, has demonstrated its excellent robustness and discriminative power in natural image classification and retrieval tasks [15,19–21]. This descriptor is usually combined with the bag-of-words (BoW) model and Fisher kernel framework to generate an image-level signature. However, its performance in retrieving brain tumor images is inferior according to the results reported by Yang [1]. Two main reasons may account for this. First, key points exist in natural images while there may be few meaningful key points existing in the brain tumor region. Second, the gradient information used in a SIFT descriptor may not have as much information as the intensity values in medical images. In view of the abovementioned analyses, we choose to use raw image patches as local features.

Several medical researchers have been engaged in CBIR. Quellec et al. [22] described a method that used wavelet transform for CBIR in medical databases. Jiang et al. [5] proposed the use of scalable image retrieval for computer-aided diagnosis of mammographic masses. Specifically, for a query mammographic region of interest (ROI), SIFT descriptors are extracted and searched in a vocabulary tree. The retrieved ROIs are used to determine whether the query ROI contains a mass. Several studies have used the BoW model to retrieve brain tumor images. In the work on brain tumor retrieval by Yang [1], the intensity profiles were extracted along the normal of tumor boundary and aggregated into a feature vector using the BoW model. Moreover, a DML algorithm, named MPP, was designed to maximize the smooth approximated mean average precision (mAP) and improve the retrieval performance. One disadvantage of Ref. [1] is that the spatial information of local features was completely disregarded. Inspired by the spatial pyramid [19], Huang et al. [2] characterized brain tumors with region-specific BoW histograms, that is, they applied BoW model to tumor region and tumor margin region separately. Densely sampled raw image patches were used as local features in their work. Compared with the work by Yang [1], the retrieval performance is improved. In another work by Huang [3], a bounding box covering the brain tumor was used as the ROI, and a learned region partition method was applied. The raw image patches were used as local features and pooled per subregion with a BoW model. A new DML algorithm aimed at minimizing rank error was adopted. Compared with Ref. [2], the retrieval performance is slightly improved.

For clarity, the specific contributions of this paper are summarized as follows:

- We augment the tumor region and use the augmented tumor region as the ROI to incorporate informative contextual information. The knowledge of the acquired medical images and disease characteristics is necessary to extract more informative features. For instance, brain tumors of the same pathological type are often found in similar places, which indicates that both tumor region and tumor-surrounding tissues can provide important clues for the identification of tumor types.
- We employ the adaptive spatial pooling strategy proposed in Ref. [23] to automatically split the ROI into subregions based on intensity orders. In this case, both spatial information and intensity distributions are considered, thereby making the final feature representations more discriminative.
- We investigate the power of the FV to retrieve brain tumor images of the same pathological type and compare it with BoW. Experimental results show that FV significantly outperform BoW. The main drawback of BoW is that it normally requires a large-sized visual vocabulary to obtain good performance, especially for datasets with large variation. The descriptor quantization is a lossy process as outlined by Boiman et al. [21]. A small vocabulary leads to large quantization error, thereby making the final feature representation less discriminative.

The rest of this paper is organized as follows. Section 2 introduces the image data and the details of each component of the proposed method. Section 3 presents the experimental results. Section 4 gives the discussion. Finally, Section 5 summarizes the conclusion of this work.

## Materials and Methods

### Ethics statement

The Ethics Committees of Nanfang Hospital and General Hospital,Tianjin Medical University approved all study procedures. Patient records/information was anonymized and de-identified prior to analysis, and written informed consent was obtained from all participants.

### Image data

The proposed method is based on 2D slices. In clinical settings, usually only a certain number of slices of brain contrast-enhanced MRI (CE-MRI) with a large slice gap are acquired and available. Therefore, the development of a 2D image-based CBIR system for clinical applications is more practical. The dataset of brain T1-weighted CE-MRI images consists of 3064 slices from 233 patients, including 708 meningiomas, 1426 gliomas, and 930 pituitary tumors, which are publicly available (http://dx.doi.org/10.6084/m9.figshare.1512427). Representative slices that have large lesion sizes are selected to construct the dataset. In each slice, the tumor boundary is manually delineated by radiologists.

### Overview of methods

A CBIR system typically consists of two phases: offline database building and online retrieval. In the offline database building phase, brain tumor images undergo a series of processing steps, including tumor segmentation, feature extraction, and distance metric learning. Features of these images, with class labels and some other meta-data associated with diagnostic information, are indexed and stored in the database. In the online retrieval phase, when given a query image, we extract the features of the query image in the same way and compare it with the image features in the database via the learned distance metric. The most similar images are returned and can be used by a radiologist to aid diagnosis.

**Fig 2:**
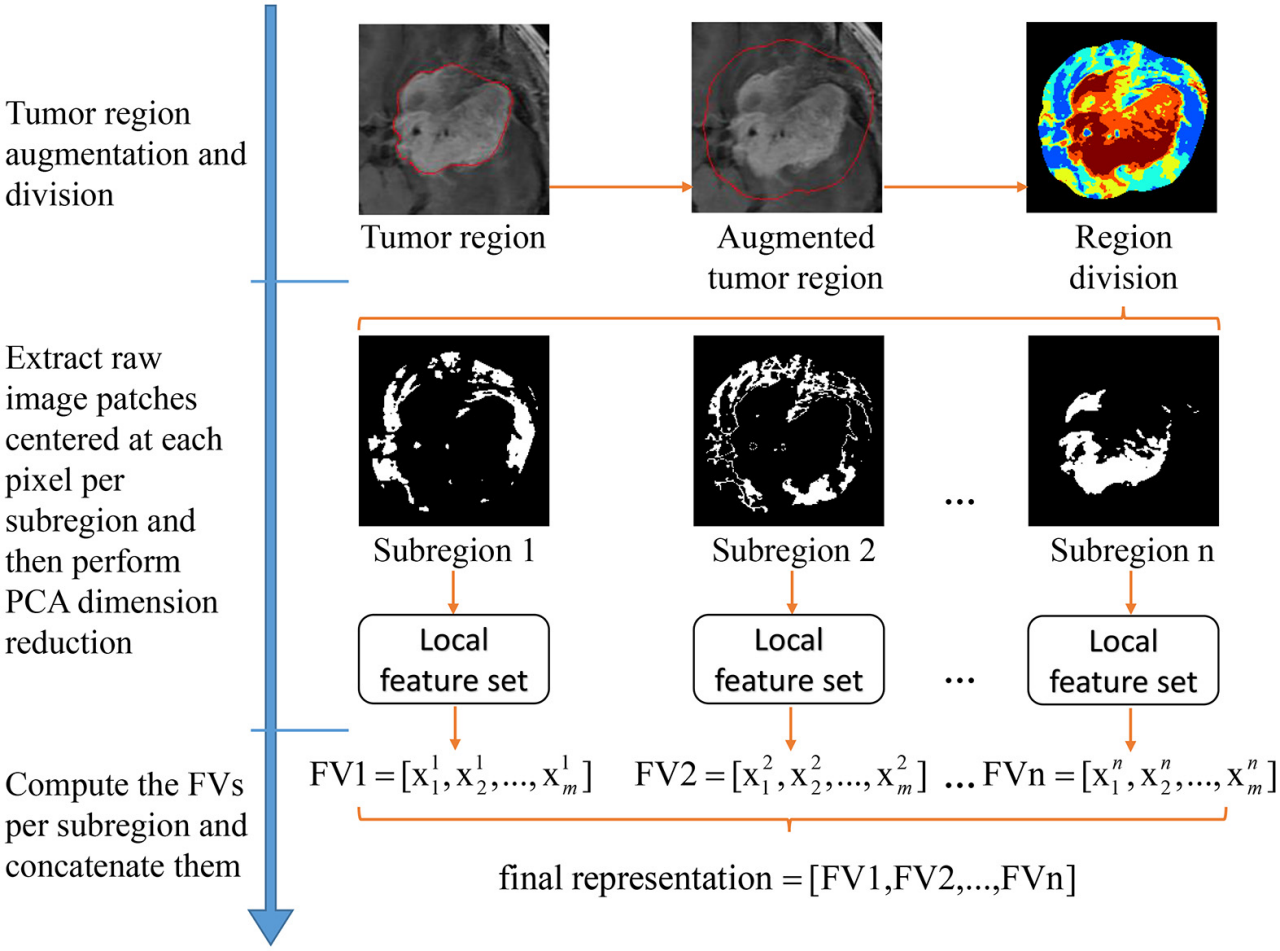
Workflow of the proposed feature extraction framework.

The three-step workflow of the proposed feature extraction framework is shown in Fig 2. First, by considering the informative contextual information, we augment the tumor region and use the augmented tumor region as the ROI (the tumor region is manually delineated in this paper, but actually we only need to roughly outline its location because we use the augmented tumor region as the ROI). Second, based on the intensity orders, the ROI is split into several subregions; within each subregion, we extract raw image patches as local features and then reduce their dimension by principal component analysis (PCA). Third, by inheriting the principle of a spatial pyramid [19], Fisher vectors (FVs) are computed per subregion; the resulting FVs are concatenated to form the final feature representation. A closed-form metric learning algorithm is adopted for distance metric learning; this algorithm is simple and effective [2,24] to measure the similarity/distance between the query image and the database images.

### Tumor region augmentation

As pointed out in Refs. [25,26] with regard to feature region detection, capturing a certain amount of context around a detected feature benefits by enlarging the descriptor measurement region. This approach can also help with the feature extraction in brain tumor images because the tissues surrounding the tumor can provide a basis for the diagnosis of the brain tumor. For example, meningiomas are usually adjacent to the skull, gray matter, and cerebrospinal fluid. Gliomas typically involve white matter. Pituitary tumors are adjacent to sphenoidal sinus, internal carotid arteries, and optic chiasma. In the work by Yang [1], the tumor margin information was leveraged by sampling the intensity profiles along the normal of tumor boundary and applying the BoW model to aggregate the intensity profiles into a feature vector. However, in this paper, we simply augment the tumor region via image dilation with a disk-shaped structuring element of radius *R* and use the augmented tumor region as the ROI. This procedure is illustrated in Fig 2. An appropriate *R* can be determined by testing several different values.

### Region division

We adopt the region division method proposed in Ref. [23] to divide the ROI into multiple subregions. Specifically, all the pixels in the ROI are first sorted by their intensities in ascending order. Subsequently, these pixels are divided equally into *n* bins, where pixels in the same bin form a subregion. Fig 2 illustrates an example of intensity order-based region division.

After region division, the raw image patches, which are centered at each pixel in each of the *n* subregions, are pooled by the Fisher kernel framework. The resulting FVs for these subregions are concatenated to form the representation of the brain tumor image.

The division based on intensity orders is not limited to the shape of the ROI. A spatial pyramid with fixed grid partitioning was introduced by Lazebnik et al. [19] to take into account the rough geometry of a scene. This approach was shown to be effective for scene recognition [19]. However, the fixed grid partitioning method is unsuitable for direct application to ROIs with large variations in shape. The ROIs used in this paper are the cases. Division based on the intensity orders naturally bypasses this problem, which is spatially adaptive and more flexible.

### Fisher vector

In this section, we provide a self-contained representation of the Fisher kernel framework [15,16], which is a powerful technique to convert a variable-size set of independent samples into a fixed-size vector representation. The Fisher kernel characterizes a sample by its deviation from the generative model. The deviation is measured by computing the gradient of the sample log-likelihood with regard to the model parameters. This approach produces a vectorial representation that we refer to as FV. In this paper, the samples are vectorized raw image patch descriptors. We choose a Gaussian mixture model (GMM) as generative model, which can be understood as a “probabilistic visual vocabulary.” Subsequently, we will describe how to compute a FV for an image.

Let *X* = {*x*_*t*_, *t* = 1…*T*} be the set of *d*-dimensional local features extracted from an image, for example, a set of image patches extracted from one subregion in this paper. The generation process of *X* can be modeled by a probability density function $u_{\lambda }=\sum_{k=1}^{K}w_{k}u_{k}$, which is a GMM of *K* components with parameters *λ* = {*w*_*k*_, *μ*_*k*_, ∑_*k*_, *k* = 1,…, *K*} where *w*_*k*_, *μ*_*k*_, and ∑_*k*_ are the mixture weight, mean vector, and diagonal covariance matrix, respectively, of Gaussian *u*_*k*_. Jaakkola and Haussler [16] proposed to represent *X* with the following vector:

$$G_{\lambda }^{X}=\frac{1}{T}\nabla_{\lambda }\text{log}u_{\lambda }(X).$$ (1)

The gradient of the log-likelihood describes how the parameters of the generative model should be modified to better fit the data *X*. Note that the dimensionality of $G_{\lambda }^{X}$ depends only on the number of parameters in *λ* and not on the sample size (*T*). A natural kernel on these gradients is the “Fisher kernel” [16]:

$$K(X,Y)=G_{\lambda }^{X}F_{\lambda }^{-1}G_{\lambda }^{Y}$$ (2)

where *F*_*λ*_ is the Fisher information matrix of *u*_*λ*_:

$$F_{\lambda }=E_{x\sim u_{\lambda }}[\nabla_{\lambda }\text{log}u_{\lambda }(x)\nabla_{\lambda }\text{log}u_{\lambda }{\left(x\right)}^{\text{T}}].$$ (3)

Given that *F*_*λ*_ is positively semi-definite; thus, *F*_*λ*_ has a Cholesky decomposition: $F_{\lambda }^{-1}=L_{\lambda }^{\text{T}}L_{\lambda }$. Therefore, the Fisher kernel *K*(*X*, *Y*) can be rewritten as a dot-product between normalized vectors (*g*_*λ*_), which are obtained as

$$g_{\lambda }^{X}=L_{\lambda }G_{\lambda }^{X}.$$ (4)

We refer to this normalized gradient vector as the FV of *X*.

Subsequently, we will give explicit expressions of the gradients. The parameters of GMM are estimated on a large training set of local features by maximum likelihood estimation. We use the diagonal closed-form approximation of the Fisher information matrix proposed in Ref. [17]. In this paper, the gradients with respect to the mean and variance are used. Let *γ*_*t*_(*k*) denote the soft assignment of *x*_*t*_ to the Gaussian *k*, which is also known as the posterior probability:

$$\gamma_{t}(k)=\frac{w_{k}u_{k}(x_{t})}{\sum_{i=1}^{K}w_{i}u_{i}(x_{t})}.$$ (5)

Let $g_{u_{k}}^{X}$ and $g_{\sigma_{k}}^{X}$ denote the *d*-dimensional gradients with respect to the mean *μ*_*k*_ and variance *σ*_*k*_, respectively, of Gaussian *k*, where *σ*_*k*_ is the variance vector, that is. the diagonal of ∑_*k*_. After standard mathematical derivations, we obtain

$$g_{u_{k}}^{X}=\frac{1}{\sqrt{w_{k}}}\sum_{t=1}^{T}\gamma_{t}(k)\left(\frac{x_{t}-u_{k}}{\sigma_{k}}\right),$$ (6)

$$g_{\sigma_{k}}^{X}=\frac{1}{\sqrt{w_{k}}}\sum_{t=1}^{T}\gamma_{t}(k)\frac{1}{\sqrt{2}}\left(\frac{{\left(x_{t}-u_{k}\right)}^{2}}{\sigma_{k}^{2}}-1\right),$$ (7)

where the division and exponentiation should be understood as term-by-term. The final FV is the concatenation of the gradients $g_{u_{k}}^{X}$ and $g_{\sigma_{k}}^{X}$ for *k* = 1,…, *K*; thus, the FV is of dimension 2*dK*. Compared with BoW, FV only needs 2*d* times fewer visual words to obtain a signature of the same length. In Section 3, experiments demonstrate that excellent results can be obtained even with a small number of visual words ranging from *K* = 16 to *K* = 128.

As mentioned in previous work [15,27], two measures are required to ensure that FV has excellent performance. The first measure is PCA dimensionality reduction; the second is normalization. Before applying the Fisher kernel framework, the dimensionality of local features is reduced by PCA. Two reasons may explain the positive effect of PCA [15]. First, PCA provides a better fit to the diagonal covariance matrix assumption because of the decorrelation effect of PCA. Second, the GMM estimation is noisy for less energetic components. Normalization consists of two steps. First, power normalization is performed to each component of the FV with the following operator:

$$f(z)=\text{sign}(z){\left|z\right|}^{\alpha }$$ (8)

where *α* is typically set to 0.5. Second, the power-normalized FV is L2-normalized.

### Closed-form metric learning

In the retrieval phase, the similarities are computed between the query image and database images. An appropriate distance metric is crucial to a CBIR system of good performance. The power of traditional rigid distance functions, such as the Euclidean distance and cosine similarity, is limited because of the complexity of the image content and the sematic gap between low-level visual features and high-level human interpretation [28,29]. To alleviate this problem, numerous distance metric learning algorithms can be applied [1,3,30–33]. The primary idea of distance metric learning is to find an optimal metric that keeps intraclass samples close while separating interclass samples as much as possible.

A major branch of metric learning is to learn the Mahalanobis distance. In literature, the (squared) Mahalanobis distance refers to the generalized quadratic distance, which is defined as:

$$\begin{array}{c} d_{M}(x_{i},x_{j})={\left(x_{i}-x_{j}\right)}^{\text{T}}M\left(x_{i}-x_{j}\right)\\ ={\left(x_{i}-x_{j}\right)}^{\text{T}}L^{\text{T}}L\left(x_{i}-x_{j}\right)\\ ={\left(Lx_{i}-Lx_{j}\right)}^{\text{T}}\left(Lx_{i}-Lx_{j}\right) \end{array}$$ (9)

where *x*_*i*_∈**R**^*n*^, *M*∈**R**^*n*×*n*^, and *M* is a positive semi-definite (PSD) matrix. From this equation, we can see that if *M* is low-rank, a linear projection of the data is induced into a space of lower dimensions. Therefore, a more compact representation of the data with cheaper computational cost is produced. Some researchers propose to optimize an objective function with respect to *M* while others perform optimization with respect to *L*. Optimization with respect to *M* often needs the projection of *M* to the PSD cone by setting the negative eigenvalues to zero at each iteration [30,33]. However this procedure is extremely time-consuming especially for high-dimensional features because of the eigen-decomposition on a large matrix. Optimization with respect to *L* can reduce the need for costly projections on the PSD cone. Based on these considerations above and the fact that the FV is typically high-dimensional, we adopt the closed-from metric learning algorithm (CFML) proposed by Alipanahi et al. [24]. The same algorithm was used by Huang [2] to retrieve brain tumor images.

Subsequently, we briefly introduce the closed-form metric learning algorithm. Suppose that the class labels are provided. We can then construct two sets of image pairs:

$$\begin{array}{l} S=\left\{\left(x_{i},x_{j})|\text{label}(x_{i})=\text{label}(x_{j}\right)\right\}\\ D=\left\{\left(x_{i},x_{j})|\text{label}(x_{i})\neq \text{label}(x_{j}\right)\right\} \end{array}$$ (10)

where label(*x*_*i*_) denotes the class label of the image representation *x*_*i*_. The optimization problem is expressed as

$$\begin{array}{l} \underset{L}{\text{arg min}}\text{ Tr}(L\left(M_{S}-M_{D}\right)L^{\text{T}})\\ \text{s}.\text{t}.\;LM_{S}L^{\text{T}}=I \end{array}$$ (11)

where

$$M_{S}=\frac{1}{\left|S\right|}\sum_{\left(x_{i},x_{j}\right)\in S}\left(x_{i}-x_{j}\right){\left(x_{i}-x_{j}\right)}^{\text{T}},$$ (12)

$$M_{D}=\frac{1}{\left|D\right|}\sum_{\left(x_{i},x_{j}\right)\in D}\left(x_{i}-x_{j}\right){\left(x_{i}-x_{j}\right)}^{\text{T}},$$ (13)

and Tr() denotes the trace of a square matrix, and *I* is an identity matrix.

The CFML algorithm tries to minimize the squared Mahalanobis distances between intraclass points while maximizing the squared Mahalanobis distances between interclass points. The closed-form solution is provided by the matrix of eigenvectors corresponding to the largest eigenvalues of the matrix $M_{S}^{-1}M_{D}$. Similar to the previous work [2,4], we also use a regularization form of CFML in this paper by replacing *LM*_*S*_*L*^T^ = *I* with *L*(*M*_*S*_+*λI*)*L*^T^ = *I* where *λ* is a small positive value that was empirically set to 1.5e-4.

## Results

### Experimental settings

Following the experimental setup in Refs. [1,3], we randomly split the 233 patients into 5 subsets of roughly equal size. Partitioning according to the patient ensures that slices from the same patient will not simultaneously appear in the training set and test set. For all the experiments, fivefold cross-validation is used. In fivefold cross-validation, one subset is sequentially used as the test set (query images), whereas the remaining four subsets are used as training set (database).

To evaluate the retrieval performance, we adopt the mAP and top-*n* retrieval precision (denoted as prec@n). We report the final results as the mean of the results of five runs. Below, we will introduce how to compute mAP and prec@n. Precision and recall can by calculated by:

$$\begin{array}{l} precision=\overset{K}{\underset{i=1}{\sum }}T_{i}/K,\\ \;\;recall=\overset{K}{\underset{i=1}{\sum }}T_{i}/\overset{N}{\underset{i=1}{\sum }}T_{i}, \end{array}$$ (14)

where *T*_*i*_ = 1 if query image *x*_*q*_ and database image *x*_*i*_ are relevant (namely they contain tumors of the same type); otherwise, *T*_*i*_ = 0, *K* = 1,2,…*N* is the number of retrieved images, and *N* is the number of images in the database. Given a query image, the images in database are ranked according to their distances to the query image in ascending order. Prec@n is the precision at the position where the n most similar database images are returned. The average precision (AP) is the average of the precisions at the positions where a relevant image exists in the ranking list. Finally, the mAP is just the mean AP over all the query images.

For the PCA dimensionality reduction on local features, we randomly choose 300 K local features from the training set to learn the PCA projection matrix. Then the dimensionality of all the local features extracted from training set and test set is reduced by multiplying the projection matrix. The reduced dimensionality is determined such that the preserved components explain at least 99% of all variability.

For FV computation, we use the released code from the VLFeat toolbox [34].

### Parameter selection

There are five parameters in our method: (1) the radius (*R*) of the disk-shaped structuring element used to dilate the tumor region, (2) the number (*N*) of pooling regions created by the intensity order-based division method, (3) the size (*W*) of raw image patches that are used as local features (i.e., *W*×*W* square patch), (4) the number (*K*) of vocabulary size (i.e., the number of Gaussians in a GMM), and (5) the reduced dimensionality (*D*) in new space induced by the projection matrix (L) learned in CFML, namely the number of rows of L. Given that trying every possible parameter combination is impractical, we choose to observe the effect of one parameter each time while keeping the other parameters fixed. In addition, we consider only the effect of the parameters *R*, *N*, and *W* in this section. The effect of parameters *K* and *D* will be presented in the subsequent section.

#### Radius of disk-shaped structuring element

We set the parameters *N*, *W*, *K*, and *D* to 1, 7, 64, and 2, respectively, and let *R* range from 0 to 32. Retrieval results with different *R* are shown in Table 1. *R* = 0 indicates we only the tumor region as the ROI without augmentation. From *R* = 0 to *R* = 8, the mAP value is significantly improved, which proves our previous statement that tumor-surrounding tissues can also provide important clues for the identification of brain tumor types. The best result is obtained at *R* = 24. When R equals 24, the performance begins to decrease, which is expected because too much normal tissues are included. In our subsequent experiments, we set *R* to 24.

**Table 1:** Evaluation of mAP performance with different *R* (mean ± std %).

*R*	0	8	16	24	32
mAP	84.01±1.68	86.31±1.74	87.74±1.46	88.33±1.36	87.88±0.97

#### Number of pooling regions

We set the parameters *R*, *W*, *K*, and *D* to 24, 7, 64, and 2, respectively, and let *N* range from 1 to 8. Table 2 shows that the retrieval performance is consistently improved with the increasing number of pooling regions. However, we do not try values greater than 16 because of the computational cost. Considering that from *R* = 8 to *R* = 16, the improvement is slight and that we will observe the effect of other parameters in the following experiments, we set *N* to 8 in the subsequent experiments.

**Table 2:** Evaluation of mAP performance with different *N* (mean ± std %).

*N*	1	2	4	8	16
mAP	88.33±1.36	90.27±0.99	92.38±0.78	93.50±0.53	94.01±0.46

#### Patch size

We set the parameters *R*, *N*, *K*, and *D* to 24, 8, 64, and 2, respectively, and let *W* range from 5 to 9. As shown in Table 3, the retrieval performance is improved as the *W* increases from 5 to 9, but the improvement from *W* = 5 to *W* = 7 is more obvious than that from *W* = 7 to *W* = 9. This difference may be attributed to the texture of larger patches, which contain more variability than that of small patches. Thus, a larger vocabulary and a larger training set of local features are needed to exert the largest effect. Section 3.3 shows that when a larger vocabulary is used, the performance can be further improved. In the following experiments, we set *W* to 9.

**Table 3:** Evaluation of mAP performance with different *W* (mean ± std %).

*W*	5	7	9
mAP	90.37±1.19	93.50±0.53	93.91±0.63

### Comparison with BoW

We compare two local feature aggregation methods: the BoW and FV representations. For the BoW representation, a *k*-means clustering algorithm is used to generate the visual vocabulary. Note that we use the same feature extraction framework for BoW and FV. That is, we use the augmented tumor region as the ROI, divide it into subregions, apply the local feature aggregation method to each subregion, and finally concatenate the per-region representations. The parameters *R*, *N*, and *W* are set to 24, 8, and 9 respectively according to Section 3.2. We let *K* range from 16 to 128, and let D range from 1 to 10. For a given vocabulary size *K*, we report the best result of different *D* values.

**Fig 3:**
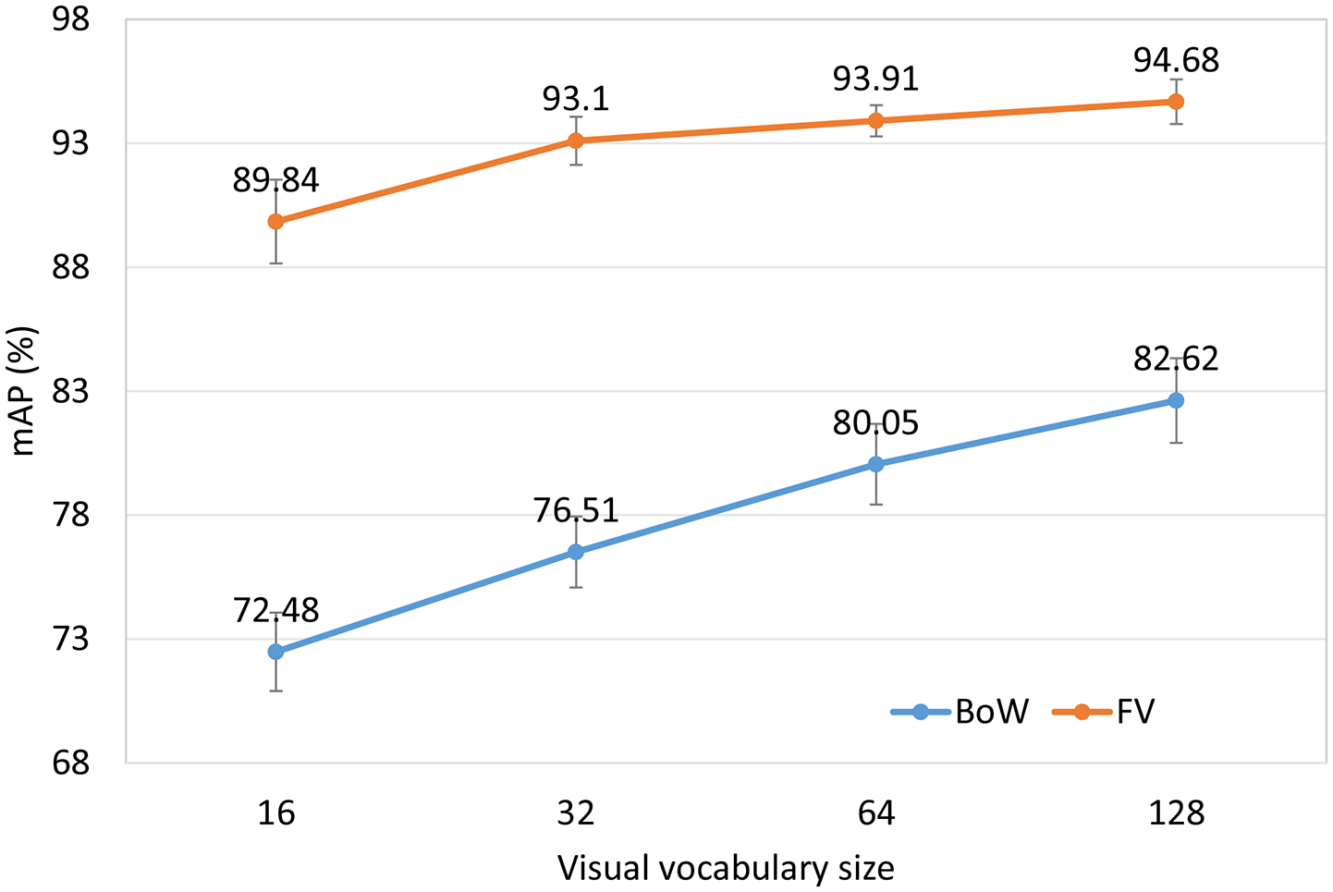
Retrieval performance of the BoW and FV as a function of vocabulary size.

**Fig 4:**
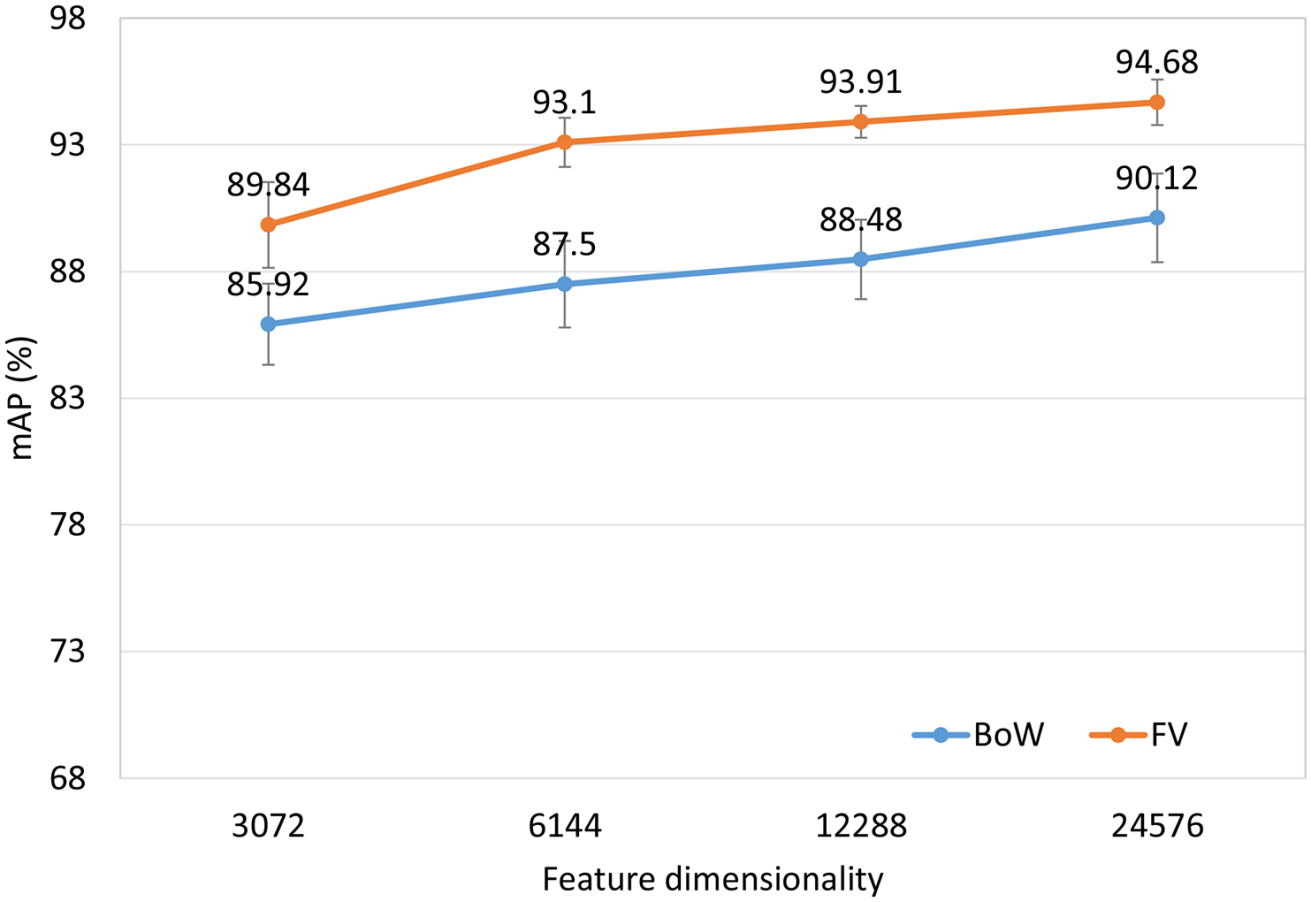
Retrieval performance of the BoW and FV as a function of feature dimensionality.

The mAP performance in Fig 3 is a function of the visual vocabulary size. Comparing BoW and FV using the same vocabulary size may be unfair to BoW since the length of FV is 2d (d is the dimensionality of the local features) times as long as BoW. We use different vocabulary size for BoW and FV to make the feature vectors of BoW and FV have the same length. Fig 4 shows the mAP performance as a function of feature dimensionality. The following observations can be made. First, as the vocabulary size or feature dimensionality increases, the retrieval performance of both BoW and FV is improved. Second, for a given vocabulary size, FV significantly outperforms BoW. This trend is to be expected because for a given vocabulary size, the dimensionality of FV is much higher than that of BoW. The difference is especially pronounced when the vocabulary size is small. Third, for a given number of dimensions, the FV also performs much better than the BoW. These results demonstrate the power of the FV representation.

**Fig 5:**
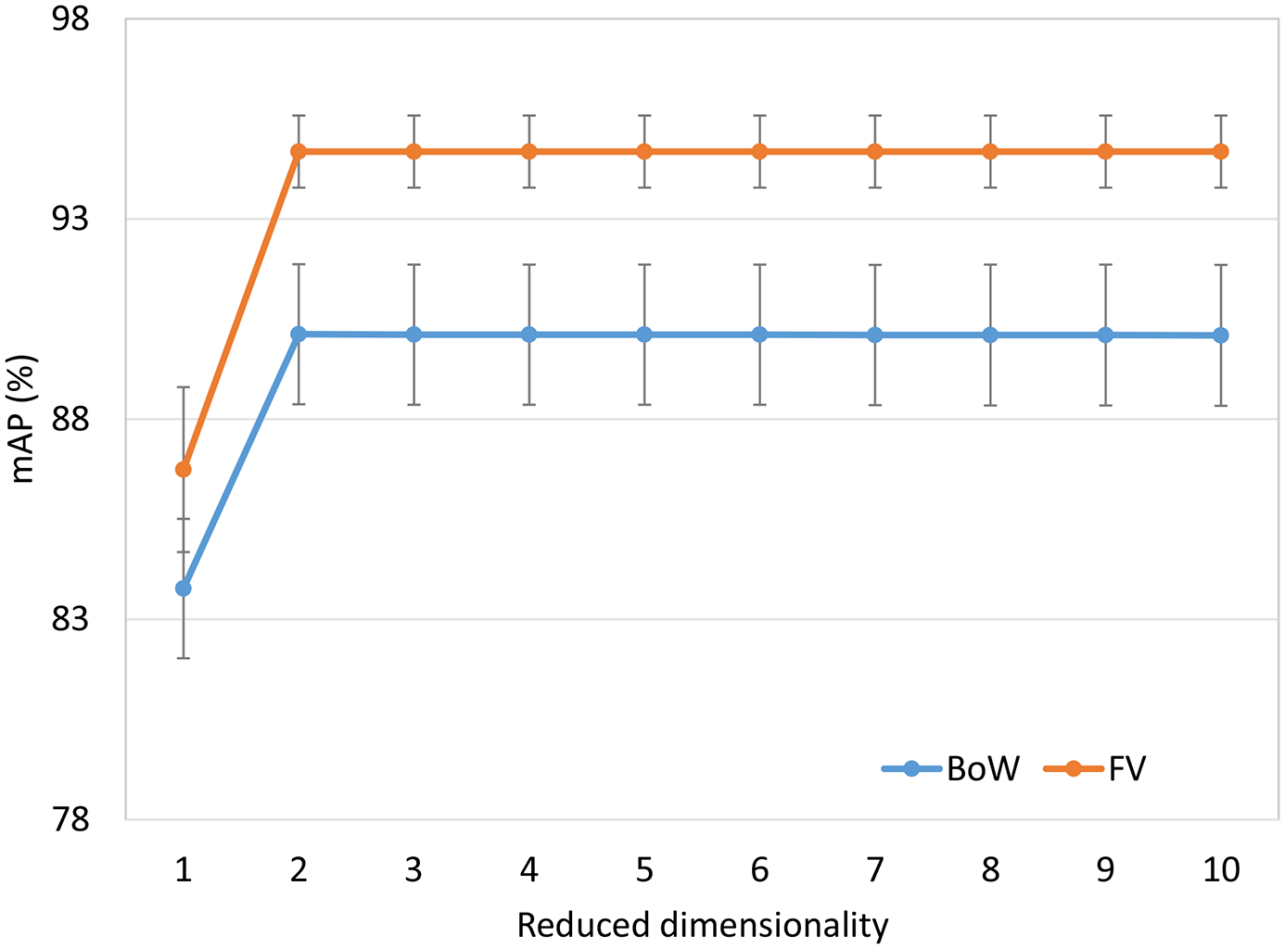
Evaluation of mAP performance with different *D* for the BoW and FV.

We also show the effect of different values of *D* on the BoW and FV in Fig 5. The feature dimensionality for BoW and FV is 24 576. As can be seen, the best results for both BoW and FV are achieved when *D* is equal to 2. When *D* is greater than 2, the mAP performance nearly remains unchanged. These results indicate that the CFML is robust for the reduced dimensionality *D*. Furthermore, achieving good retrieval performance at such low dimensionality can significantly reduce the computational cost in the retrieval phase. In our experiments, we observed the effect of parameter *D* for other feature dimensionalities has similar curves, so we list only one example for brevity here.

### Retrieval performance of the proposed method

Compared with the mixed-type retrieval performance, we evaluate the retrieval performance of the proposed method for different tumor types in this section. The parameters *R*, *N*, *W*, *K*, and, *D* are set to 24, 8, 9, 128, and 2, respectively. Table 4 summarizes the results. The retrieval performance of meningiomas is much lower than those of gliomas and pituitary tumors. One possible reason is the data imbalance between different tumor categories.

**Table 4:** Retrieval performance of the proposed method for different types of brain tumors (mean ± std %).

Tumor type	mAP	Prec@10	Prec@20
Meningioma	88.77±3.07	86.33±4.04	86.30±3.96
Glioma	97.64±0.67	95.98±0.96	96.05±1.01
Pituitary tumor	94.82±3.42	92.76±4.69	92.77±4.68

### Comparison with related works

To demonstrate the power of the proposed method, we compare it with three other brain tumor retrieval methods [1–3]. A brief overview of the three methods can be found in Section 1. In all four methods, the same dataset is used with fivefold cross-validation. The comparison results are shown in Table 5. The retrieval results of the three compared methods are directly extracted from the corresponding original papers. The mAP of our method significantly outperforms those of the other three methods.

**Table 5:** Comparison of our method with three related methods (%).

Methods	Yang et al. [1]	Huang et al. [2]	Huang et al. [3]	Ours
mAP	87.3	91.0	91.8	**94.68**

## Discussion

In this paper, we address the problem of retrieving brain tumor images in archives that have the same pathological type as the query image. The retrieved images with diagnostic information can be used by radiologists to provide decision support. The success of image retrieval systems heavily relies on good feature representations and suitable distance metrics. The effectiveness of the three components of our feature extraction method is demonstrated in our experiments. Besides, instead of using traditional rigid distance functions like Euclidean distance, a suitable distance metric is indispensable to obtain good retrieval performance. For example, using CFML, we can achieve a mAP as high as 94.68%, while replacing the learned distance metric with Euclidean distance we obtained a mAP of 59.64%.

In addition, the best results with our method are obtained when we apply the CFML algorithm to project the feature representations into a new space of two dimensions. This feature is beneficial for computational and memory efficiency. Another potential advantage is that low-dimensional feature vectors can facilitate the indexing techniques (e.g., KD-tree, R-tree, R*-tree, and quad trees [35]) for a large-scale database. The indexing techniques only compare the query image with a portion of the database images to improve the retrieval efficiency. However, the performance of all these indexing structures is reduced by high-dimensional feature vectors.

Future endeavors to improve the CBIR system for brain tumor retrieval will be devoted to the following two aspects. First, semiautomatic or fully automatic methods can be integrated into the retrieval system to reduce the workload of the radiologists although the tumor region does not need precise segmentation in this paper. Second, multiple types of features, such as intensity, texture, shape, BoW, and FV, can be utilized. To this end, one possible solution is to simply concatenate all these features. However, this naive concatenation approach may suffer from two drawbacks: (1) some types of features may significantly dominate the others in the DML task; thus, the potential of all features cannot be fully exploited; (2) the resulting high-dimensional feature space will make the subsequent DML task computationally expensive. To overcome these drawbacks, we can employ the scheme of multi-modal DML [36], which learns to optimize a separate distance metric for each type of feature space and simultaneously learns to find optimal combination weights of different distance metrics on multiple types of feature space.

## Conclusion

In this paper, a new CBIR system for retrieving three types of brain tumors (meningiomas, gliomas and pituitary tumors) in T1-weighted contrast-enhanced MRI images is presented, which can serve as a tool for computer-aided diagnosis. Using the augmented tumor region as ROI, we find that tumor-surrounding tissues can provide valuable information for brain tumor categories. An intensity orders based region division method is applied to make the feature representations more discriminative, which can capture both spatial information and intensity distributions. Finally we use the powerful FV to aggregate local features of each subregion into a feature vector. The performance of the proposed CBIR system provides a substantial improvement against three closely related methods, achieving a mAP of 94.68%.
